# Biochemical and Structural Insights into a Novel Thermostable β-1,3-Galactosidase from *Marinomonas* sp. BSi20414

**DOI:** 10.3390/md15010013

**Published:** 2017-01-08

**Authors:** Haitao Ding, Qian Zeng, Lili Zhou, Yong Yu, Bo Chen

**Affiliations:** SOA Key Laboratory for Polar Science, Polar Research Institute of China, Shanghai 200136, China; htding@outlook.com (H.D.); zengqianmu@126.com (Q.Z.); lilizhou1199@163.com (L.Z.); yuyong@pric.org.cn (Y.Y.)

**Keywords:** β-galactosidase, *Marinomonas*, thermostable, purification, gene cloning, linkage selectivity

## Abstract

A novel β-1,3-galactosidase, designated as MaBGA (β-galactosidase from *Marinomonas* sp. BSi20414), was successfully purified to homogeneity from *Marinomonas* sp. BSi20414 isolated from Arctic sea ice by ammonium sulfate precipitation and anion exchange chromatography, resulting in an 8.12-fold increase in specific activity and 9.9% recovery in total activity. MaBGA displayed its maximum activity at pH 6.0 and 60 °C, and maintained at least 90% of its initial activity over the pH range of 5.0–8.0 after incubating for 1 h. It also exhibited considerable thermal stability, which retained 76% of its initial activity after incubating at 50 °C for 6 h. In contrast to other β-galactosidases, MaBGA displayed strict substrate specificity, not only for the glycosyl group, but also for the linkage type. To better understand the structure–function relationship, the encoding gene of MaBGA was obtained and subject to bioinformatics analysis. Multiple alignments and phylogenetic analysis revealed that MaBGA belonged to the glycoside hydrolase family 42 and had closer genetic relationships with thermophilic β-galactosidases of extremophiles. With the aid of homology modeling and molecular docking, we proposed a reasonable explanation for the linkage selectivity of MaBGA from a structural perspective. On account of the robust stability and 1,3-linkage selectivity, MaBGA would be a promising candidate in the biosynthesis of galacto-oligosaccharide with β1–3 linkage.

## 1. Introduction

The enzyme β-galactosidases (EC 3.2.1.23, BGA), which are widely distributed in various organisms, including animals, plants, bacteria, archaea, yeasts and fungi, are capable of catalyzing the hydrolysis of molecules containing the β-glycosidic bond, to release their terminal non-reducing galactose molecules. In some cases, β-galactosidases can catalyze the reverse reaction of the hydrolysis, transglycosylation, when receptors of galactosyl are monosaccharides, disaccharides or oligosaccharides, instead of water molecules [1]. Due to the catalytic characteristic, β-galactosidases are important for the dairy industry to produce milk with low/no lactose for people who suffer from lactose intolerance [2]. Moreover, β-galactosidases are also widely utilized for enzymatic synthesis of galacto-oligosaccharides, which can be employed to stimulate the growth of beneficial bacteria selectively in the gut, as prebiotics [3].

Based on the similarity of the amino acid sequences, β-galactosidases are mainly divided into four glycoside hydrolase (GH) families [4]—GH1, GH2, GH35 and GH42—according to the carbohydrate-active enzymes database (CAZy) [5]. All of these four families belong to the GH-A superfamily, of which members have two glutamic acid residues as catalytic active sites located in an (α/β)_8_ TIM (the triosephosphate isomerase) barrel domain [6]. Generally, GH1 and GH2 β-galactosidases are mainly found in mesophiles and display high lactase activity [7]. GH35 β-galactosidases are usually found in pathogens such as Streptococcus pneumoniae [8,9,10], with specific activity toward β-1,3-linkages. β-galactosidases belonging to the GH42 family are mostly stemmed from extremophiles, including thermophiles [11,12,13,14,15], halophiles [16,17] and alkaliphiles [18].

Owing to the attractive properties such as heat resistance and salt tolerance, GH42 β-galactosidases have received extensive attention in recent years. It is expected to obtain new enzymes with excellent properties from microorganisms living in extreme environments [19]. The Arctic is one of the most extreme regions to be inhabited by plenty of microorganisms, which have been proven to be the natural treasure house for screening novel enzymes [20,21]. In our previous study, a strain designated as BSi20414 with high β-galactosidase activity was isolated from Arctic sea ice and identified as *Marinomonas* [22]. The optimal catalytic temperature of the crude enzyme was determined as 60 °C, indicating that it might be a thermophilic enzyme. Generally, robust thermal-stability is indispensable for the practical application of enzymes. Thus, to obtain a promising thermal-stable β-galactosidase and provide a comprehensive evaluation of its potential in practical application, the enzyme that possessed β-galactosidase activity from *Marinomonas* sp. BSi20414 was purified to homogeneity and characterized extensively in the present work. In addition to biochemical characterization, the encoding gene of MaBGA was cloned by degenerate PCR and chromosome walking, and was further subject to bioinformatics analysis to investigate its structure–function relationships.

## 2. Results

### 2.1. Purification of Wild-Type MaBGA

**Figure 1:**
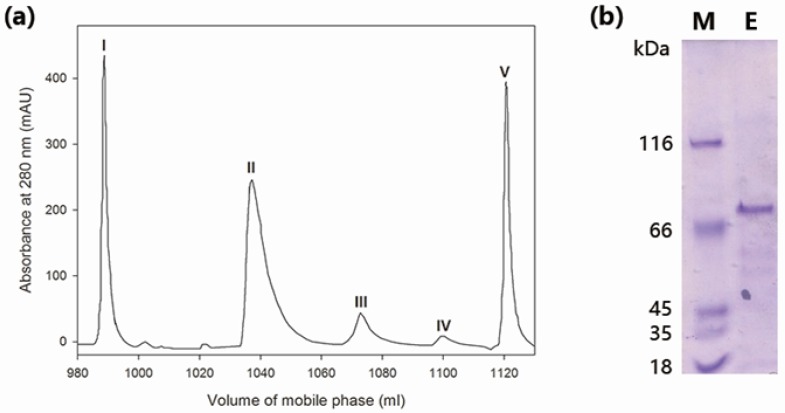
Purification of wild-type MaBGA (β-galactosidase from *Marinomonas* sp. BSi20414). (**a**) Ion exchange chromatography. Peak I was unbound proteins; Peak II and III were proteins eluted by 0.1–0.2 M of NaCl; Peak IV was protein eluted by 0.20–0.24 M of NaCl; Peak V was protein eluted by 0.24–0.6 M of NaCl; (**b**) SDS-PAGE (sodium dodecyl sulfate polyacrylamide gel electrophoresis) analysis of purified MaBGA. Lane M: protein molecular weight marker; Lane E: purified MaBGA.

The crude enzyme was concentrated by 60% of ammonium sulfate and then separated into five components, peak I–V (Figure 1a), by anion exchange chromatography. Among these five peaks, only peak IV exhibited β-galactosidase activity toward *o*-nitrophenyl-β-galactoside (ONPG). The purity of peak IV was examined by SDS-PAGE (sodium dodecyl sulfate polyacrylamide gel electrophoresis) analysis, which showed a single band corresponding to about 70 kDa (Figure 1b), indicating that MaBGA had been successfully purified. As shown in Table 1, the two-step purification procedure yielded an 8.12-fold increase in specific activity and a recovery of 9.9% in total activity.

### 2.2. Enzymatic Characterization of MaBGA

#### 2.2.1. Effect of pH on the Activity and Stability of MaBGA

**Figure 2:**
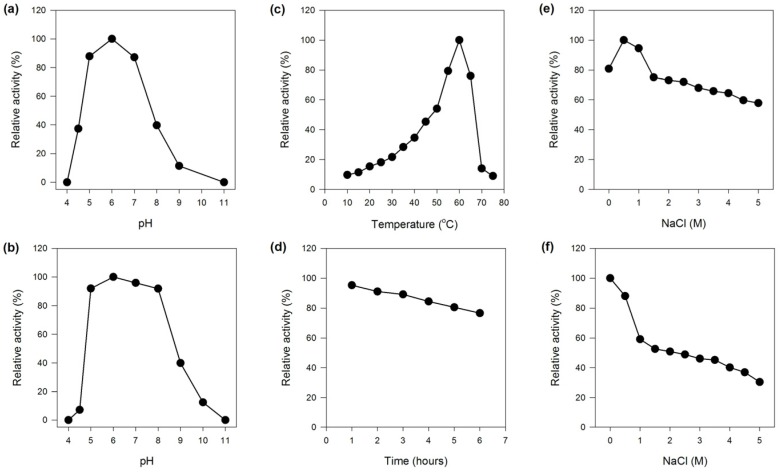
Effects of pH, temperature and NaCl on the activity and stability of MaBGA. (**a**) Effect of pH on the activity of MaBGA; (**b**) Effect of pH on the stability of MaBGA; (**c**) Effect of temperature on the activity of MaBGA; (**d**) Effect of time on the stability of MaBGA; (**e**) Effect of NaCl on the activity of MaBGA; (**f**) Effect of NaCl on the stability of MaBGA.

The optimum pH of MaBGA was determined as 6.0, and it exhibited more than 80% of its maximum activity over the pH range of 5.0–7.0, outside of which the activity decreased sharply (Figure 2a). The stability of MaBGA showed a similar pattern with that of the activity response to pH, which was stable around the neural condition, and could maintain at least 90% of its initial activity over the pH ranging from 5.0 to 8.0, after incubating in Britton–Robinson buffer with different pH values for 1 h (Figure 2b).

#### 2.2.2. Effect of Temperature on the Activity and Stability of MaBGA

MaBGA exhibited the highest activity at 60 °C, and less than 50% of the maximum activity was measured at temperatures below 45 °C (Figure 2c). Generally, an enzyme with a relatively high optimal reaction temperature often possessed superior thermal stability. With no exception, MaBGA was stable at 50 °C, which could maintain 76% of its initial activity after incubating for 6 h (Figure 2d). In addition, the half-life of MaBGA at 50 °C was determined as 16 h.

#### 2.2.3. Effect of NaCl on the Activity and Stability of MaBGA

MaBGA showed the highest activity with 0.5 M NaCl contained in the reaction buffer. Although the activity decreased along with the increase in the concentration of NaCl, MaBGA still displayed 55% of its maximum activity with 5 M NaCl added (Figure 2e). MaBGA was unstable while incubated in buffers containing NaCl above 0.5 M, and it could only maintain 30% of its initial activity after incubating in buffer with 5 M NaCl added for 1 h (Figure 2f).

#### 2.2.4. Effects of Metal Ions and Chemicals on the Activity of MaBGA

As shown in Table 2, K^+^, Na^+^ and Mn^2+^ displayed no significant effects on the activity of MaBGA, as well as EDTA. Interestingly, Fe^2+^ is capable of improving the activity of MaBGA by 111%, whereas other bivalent cations—Mg^2+^, Co^2+^, Ni^2+^ and Zn^2+^—slightly inhibited the activity of the enzyme. Moreover, reducing agents, such as l-cysteine, l-glutathion and dithiotreitol showed no notable effect on the activity of MaBGA, indicating that no disulfide bond was indispensable to the enzyme.

#### 2.2.5. Substrate Specificity and Steady-State Kinetic Analysis

MaBGA possessed a narrow substrate spectrum, which showed no activity toward *p*-nitrophenyl-β-d-glucopyranoside, *p*-nitrophenyl-β-d-xylopyranoside, *p*-nitrophenyl-β-d-lactopyranoside, *p*-nitrophenyl-β-d-glucuronide, *p*-nitrophenyl-a-d-galactopyranoside, *p*-nitrophenyl-β-l-arabinopyranoside and *p*-nitrophenyl-β-d-cellobioside. Moreover, MaBGA showed not only group selectivity, but also showed linkage selectivity in the substrate recognition process, of which the activity toward *p*-nitrophenyl-β-d-galactopyranoside was 4.22-fold greater than that of ONPG (Table 3).

The steady-state kinetic constants of MaBGA were determined by using a nonlinear fitting plot. The apparent Michaelis–Menten constant *K_m_* and the maximum reaction velocity *V*_max_ were calculated as 14.19 mM and 1.049 μM·min^−1^, respectively.

#### 2.2.6. Linkage Selectivity Analysis

**Figure 3:**
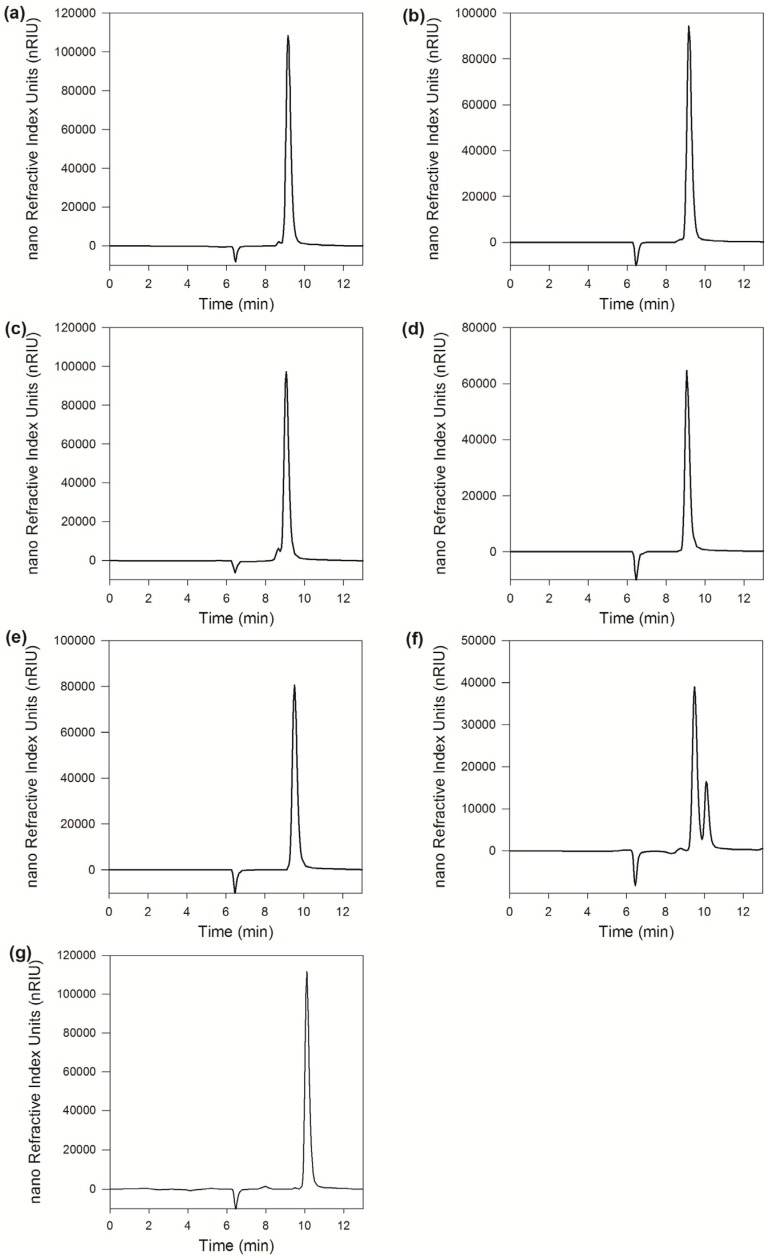
Linkage selectivity analysis. (**a**), (**c**), (**e**) and (**g**) were chromatograms of Galβ1–4GlcNAc, Galβ1–6GlcNAc, Galβ1–3GlcNAc and galactose, respectively; (**b**), (**d**) and (**f**) were chromatograms of Galβ1–4GlcNAc, Galβ1–6GlcNAc and Galβ1–3GlcNAc hydrolyzed by MaBGA, respectively.

As shown in Figure 3, the chromatograms of Galβ1–4GlcNAc showed no change before and after the reaction catalyzed by MaBGA, as well as Galβ1–6GlcNAc, suggesting that MaBGA was unable to hydrolyze both Galβ1–4GlcNAc and Galβ1–6GlcNAc. With regard to Galβ1–3GlcNAc, the product chromatogram generated a new peak corresponding to the standard of β-galactose, with an identical retention time of 10.1 min, indicating that MaBGA was capable of degrading Galβ1–3GlcNAc selectively.

### 2.3. Gene Cloning and Sequence Analysis

#### 2.3.1. Gene Cloning

**Figure 4:**
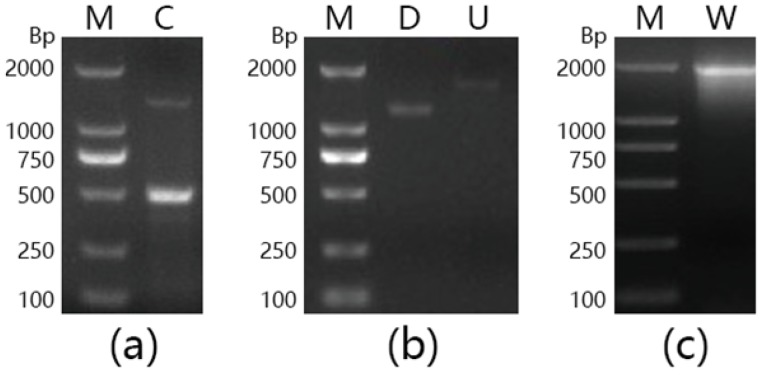
Agarose gel electrophoresis analysis of DNA fragments amplified by PCR. (**a**) Degenerated PCR; (**b**) Chromosome walking PCR; (**c**) Full length amplification. Lane M: DNA marker; Lane C: Fragment amplified by F1/R2; Lane D: Downstream fragment; Lane U: Upstream fragment; Lane W: Full-length fragment of gene *mabga*.

A 500-bp fragment was amplified from the genomic DNA of *Marinomonas* sp. BSi20414, by using the degenerate primers F1 and R2 (Figure 4a). The nucleotide sequence of the fragment showed an identity of 84% with a putative β-galactosidase gene of *Marinomonas* sp. MWYL1, suggesting that the partial β-galactosidase gene sequence of *Marinomonas* sp. BSi20414 had been successfully obtained. After chromosome walking, 1800-bp and a 1600-bp DNA fragments (Figure 4b), corresponding to the upstream and downstream sequence of the target gene, were amplified and sequenced. The intermediate and flanking sequences were utilized for assembly and subsequent amplification of the intact *mabga* gene, with a size of 2000 bp (Figure 4c).

#### 2.3.2. Sequence Analysis

Gene *mabga* encodes a peptide consisting of 656 amino acids with a calculated molecular weight of 74.28 kDa in accordance with the results of SDS-PAGE. Significant Pfam-A matches revealed that MaBGA belonged to the glycoside hydrolase family 42. The protein sequence of MaBGA showed 54.6%, 54.0%, 52.5%, 52.0%, 40.6%, and 38.2% identities with the well-characterized galactosidases from *Thermus* sp. IB-21 (Q8GEA9) [13], *Thermus thermophilus* A4 (O69315) [23], *Thermus* sp. T2 (O54315) [14], *Thermus brockianus* ITI360 (Q9X6C6) [24], *Halorubrum lacusprofundi* ATCC 49239 (B9LW38) [16], and *Haloferax lucentense* DSM 14919 (P94804) [17], respectively. In addition, MaBGA exhibited the highest identity of 90% with a putative BGA from *Marinomonas* sp. MWYL1.

**Figure 5:**
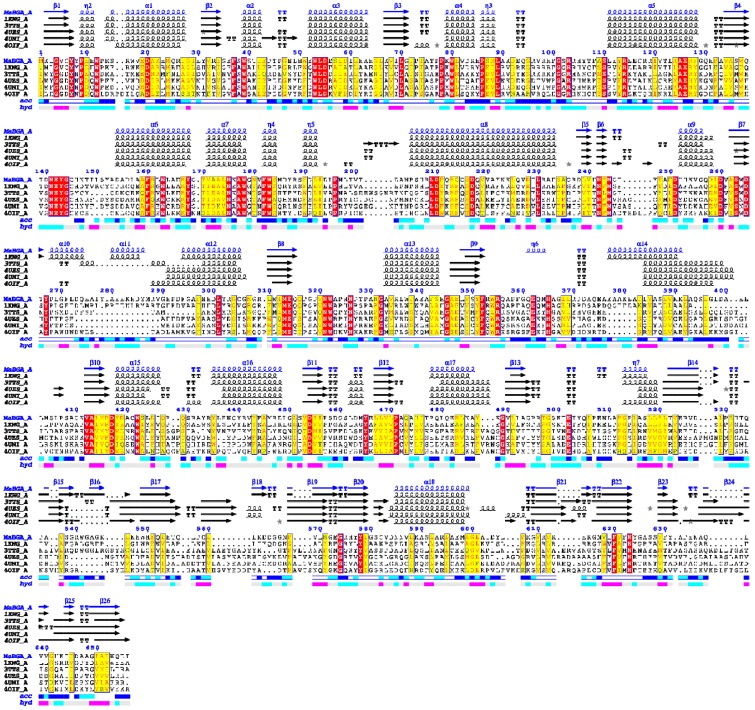
Multiple alignment of structure-solved β-galactosidases of the GH42 family (glycoside hydrolase 42 family). Identical residues and conserved substitutions are shaded red and yellow, respectively. Secondary structures of β-galactosidases are presented on the top: helices with squiggles, β-strands with arrows, turns with TT letters.

**Figure 6:**
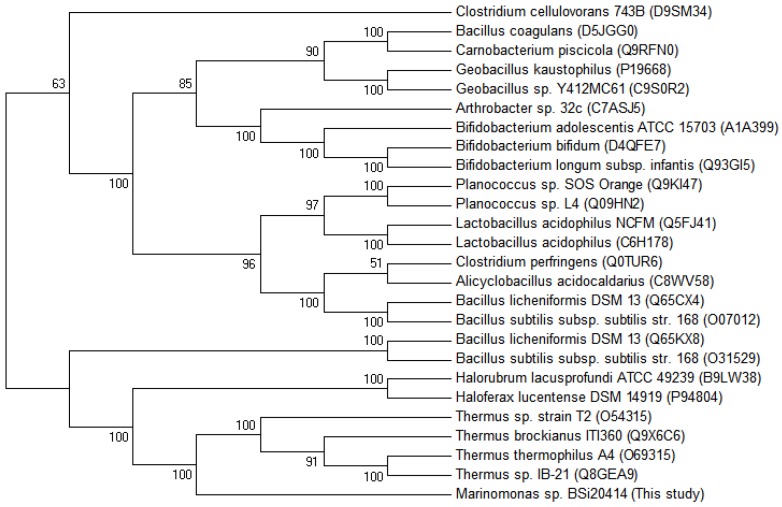
Unrooted phylogenetic tree of β-galactosidase belonging to the GH42 family. The phylogenetic tree was built using the neighbor joining method [31] in MEGA 6 [32], with a bootstrap test of 1000 replicates. The GenBank accession numbers were provided in the bracket followed by the species names.

Multiple alignments of protein sequences of structure-solved GH42 β-galactosidase showed that MaBGA shared the conserved catalytic residues, Glu142 and Glu314, as well as other GH42 BGAs (Figure 5). Phylogenetic analysis of characterized BGAs showed that these BGAs diverged into two clusters, and MaBGA affiliated to the branch consisting of thermophilic BGAs (Figure 6) that exhibited considerable stability against heat in previous studies, suggesting that these BGAs, including MaBGA, might be originated from the same ancestral sequence.

### 2.4. Structural Analysis of MaBGA

#### 2.4.1. Homology Modelling

**Figure 7:**
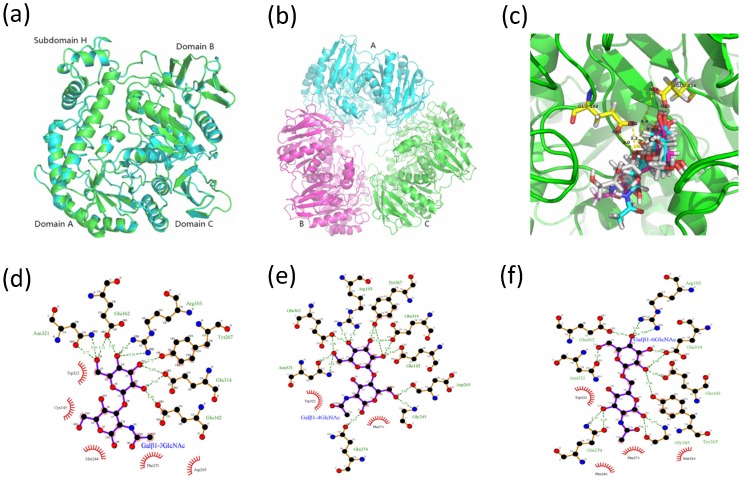
Three-dimensional structures of MaBGA. (**a**) Superposition of the MaBGA monomer structure (green) on the structures of β-galactosidase from Thermus thermophilus A4 (cyan; PDB entry 1kwk); (**b**) Ribbon representation of the trimer structure of MaBGA; (**c**) Ball and stick representation of the docking models of MaBGA with Galβ1–3GlcNAc (white), Galβ1–4GlcNAc (cyan) and Galβ1–6GlcNAc (magenta), respectively; (**d**) Schematic diagram of MaBGA/Galβ1–3GlcNAc interactions; (**e**) Schematic diagram of MaBGA/Galβ1–4GlcNAc interactions; (**f**) Schematic diagram of MaBGA/Galβ1–6GlcNAc interactions.

The tertiary structures of MaBGA were constructed by various software or online servers, including SWISS-MODEL [25], Robetta [26], MODELLER [27] and I-TASSER [28], then evaluated by ProSA [29] and PROCHECK [30]. Both of the Z-score and Ramachandran plot statistics indicated that the three-dimensional structures of MaBGA had been modeled reasonably (Table 4), especially for the model constructed by MODELLER, which scored highest and was selected for the docking study. The superposition of the MaBGA monomer structure onto the structure of β-galactosidase from *Thermus thermophilus* A4 [23] demonstrated the relatively high similarity between them, with a root mean square deviation value of 0.17 (Figure 7a). As with other GH42 BGAs [11,18,23], the quaternary structure of MaBGA was predicted as a homo-trimer, which resembles a flowerpot, with a cone-shaped tunnel in the center of the flowerpot surrounded by three subunits (Figure 7b).

#### 2.4.2. Molecular Docking Analysis

The model of MaBGA generated by MODELLER [27] was subject to GROMACS [33] software packages for energy minimization, to remove steric clashes. The refined model was employed for molecular docking with Galβ1–3GlcNAc, Galβ1–4GlcNAc and Galβ1–6GlcNAc by Autodock 4.2 [34], respectively. Cluster analysis was performed on different conformations with a root mean square deviation (RMSD) tolerance of 2.0 Å. Conformation with the lowest estimated binding free energy was utilized for analysis. As shown in Figure 7c, the galactosyl group of these three substrates adopts similar conformations, including the oxygen atom which links the acetylglucosamine group. However, the acetylglucosamine group of the substrates adopts a varied conformation corresponding to their lowest free energy. The two-dimensional projection of the interaction of the enzyme/substrate complex showed that no hydrogen bond was generated between the enzyme and the glucosyl group of Galβ1–3GlcNAc (Figure 7d), in contrast to those of Galβ1–4GlcNAc (Figure 7e) and Galβ1–6GlcNAc (Figure 7f), which formed three and four pairs with the enzyme, respectively.

## 3. Discussion

In the present study, a thermostable β-1,3-galactosidase MaBGA was successfully purified to homogeneity from *Marinomonas* sp. BSi20414 isolated from Arctic sea ice by ammonium sulfate precipitation and anion exchange chromatography, resulting in an 8.12-fold increase in specific activity and 9.9% recovery in total activity. The purification results showed that the two-step purification method is efficient for separating MaBGA from the wild-type strain of *Marinomonas* sp. BSi20414, which also provides a reference for extracting other proteins from strains belonging to the genus of *Marinomonas*.

Interestingly, as an enzyme stemmed from a strain living in permanently low-temperature marine environments, MaBGA displayed extraordinary stability against heat, with the half-life determined as 16 h at 50 °C. Phylogenetic analysis of characterized GH42 BGAs also revealed that MaBGA had closer genetic relationships with thermophilic BGAs derived from extremophiles, including thermophiles [11,12,13,14,15] and halophiles [16,17]. On account of the enzymatic and phylogenetic analyses, MaBGA was considered as a thermophilic enzyme, although the thermal stability of MaBGA is weaker than those of its thermophilic counterparts. Additionally, MaBGA only shared high identity (>70%) with BGAs of the genus *Marinomonas*, and no sequence with identity more than 55% was found in their related marine species. On the basis of the above evidence, a putative explanation was proposed to illustrate the mismatch of enzyme stability and circumstance. It is supposed that the ancestor of the genus *Marinomonas* acquired the gene encoding thermophilic β-galactosidase from other thermophiles by occasional horizontal transfer, then experienced adaptive evolution under low-temperature marine environments for a long period, which led to a decrease in thermal stability without selection pressures.

Another point worth mentioning is that MaBGA has a strict substrate specificity, unlike other GH42 BGAs. Furthermore, it displayed not only group selectivity, but also linkage selectivity in the substrate recognition process. As indicated above, MaBGA was able to hydrolyze Galβ1–3GlcNAc, but was unable to hydrolyze Galβ1–4GlcNAc and Galβ1–6GlcNAc. To better understand the linkage selectivity of MaBGA, it is essential to put MaBGA against its structural contexts. Thus, the three-dimensional structure of MaBGA was constructed and subject to docking analysis after energy minimization by molecular dynamics. As shown in Figure 7c, for all these three substrates, although the galactosyl group adopts similar lowest energy conformations, the distance is a bit long for the reaction between the oxygen atom linking the acetylglucosamine group and the carboxyl group of catalytic residues (Glu142/Glu314). Therefore, the substrate molecule needs to fine-tune its geometry to shorten the distance mentioned above by overcoming the energy barrier. However, the planar representation of the interaction of the enzyme/substrate complex indicated that the strong interaction between the glucosyl group of Galβ1–4GlcNAc (Figure 7e)/Galβ1–6GlcNAc (Figure 7f) and the enzyme might lead to the failure of the substrates to adjust their conformation for an optimal fit. Therefore, we proposed that the favored binding conformation with lowest free binding energy of the substrate is not close enough to the catalytic residues to let the reaction occur, thus the substrate might be fine-tuning its conformation to achieve an optimal geometry for the reaction. However, due to the different binding energy between the glucosyl group and enzyme, Galβ1–4GlcNAc and Galβ1–6GlcNAc cannot readily overcome the energy barrier, other than Galβ1–3GlcNAc. In general, further experiments, such as enzyme/substrate complex co-crystallization and site-directed mutagenesis, are still needed to test the hypothesis.

A previous study had proven that galacto-oligosaccharides with β1–3 linkage have a stronger bifidogenic effect than those with β1–4 and β1–6 linkages [35], indicating that the former would be more popular as prebiotics than the latter two. Generally, the production of galacto-oligosaccharides is implemented by the transglycosylation activity of β-galactosidase [36], therefore, the linkage of galacto-oligosaccharides will depend on the linkage recognition ability of β-galactosidase. Since β-galactosidases that existed in the nature which are capable of recognizing β1–3 galactoside linkage are very few, the β-1,3-galactosidase MaBGA studied in the present work not only could provide a promising candidate for the biosynthesis of galacto-oligosaccharides with β1–3 linkage, but also would offer a good model for research on the substrate recognition mechanism of β-galactosidase.

## 4. Materials and Methods

### 4.1. Strains, Plasmids, and Culture Conditions

*Strain* BSi20414, used as the source of β-galactosidase, was isolated from a core sample of sea ice collected from Canada Basin, Arctic Ocean, and was characterized as *Marinomonas* in our previous study [22]. The strain was cultivated in medium (pH 7.0) containing MgCl_2_ (0.5%, *w*/*v*), MgSO_4_·7H_2_O (0.4%, *w*/*v*), KCl (0.1%, *w*/*v*), CaCl_2_ (0.06%, *w*/*v*), lactose (1.5%, *w*/*v*) and Tryptone (0.5%, *w*/*v*), on a shaking incubator at 180 rev·min^−1^ at 30 °C for 96 h. *Escherichia coli* DH5α used for gene cloning was cultivated at 37 °C in Luria–Bertani medium. Plasmid pMD18-T (Takara) was used to conduct TA cloning for sequencing. All chemicals used in this study were of analytical grade.

### 4.2. Purification of Wild-Type MaBGA

All purification steps were conducted at 4 °C. Cells were harvested by centrifugation at 10,000× *g* for 10 min. The pellet was washed three times with normal saline and was suspended by pre-cooling PBS buffer (pH 7.0, 50 mM). The suspension was lysed by sonication (burst of 2 s followed by intervals of 5 s for 30 min). The cell debris was removed by centrifugation at 10,000× *g* for 15 min and the supernatant was precipitated with ammonium sulfate (60%, *w*/*v*). The precipitate was collected by centrifugation at 10,000× *g* for 10 min, then dissolved and dialyzed using PBS buffer (pH 7.0, 50 mM) overnight. Subsequently, the protein solution was filtered by cellulose acetate film with pore size of 0.22 μm, and the filtrate was loaded onto an anion exchange column HiTrap DEAE FF, which was pre-equilibrated with PBS buffer (pH 8.0, 50 mM). The column was first washed with PBS buffer (pH 8.0, 50 mM) for tenfold resin volumes, then was eluted by PBS buffer (pH 8.0, 50 mM) containing NaCl with a linear gradient from 0.1 M to 0.6 M. Every eluting peak was collected and measured by standard activity assay. The protein concentration was assayed by the method of Bradford using BSA (bovine serum albumin) as a standard [37].

### 4.3. SDS-PAGE Analysis

The purified MaBGA was analyzed by denaturing discontinuous SDS-PAGE on a 5% stacking gel and a 10% separating gel as described by Laemmli [38]. Gels were stained with Coomassie Brilliant Blue R-250. The molecular weight of MaBGA was determined by comparing its electrophoretic mobility with Protein Molecular Weight Marker (MBI).

### 4.4. β-galactosidase Activity Assay

The β-galactosidase activity was assayed by measuring the absorbance of ONP (*o*-nitrophenyl) at 420 nm in 50 mM PBS buffer (pH 7.0) with 10 mM ONPG as substrate. The ONP concentration was calculated from the standard curve obtained under the same experimental condition. One unit of enzyme activity was defined as the amount of the enzyme that catalyzed the formation of 1 μmol of ONP per minute.

### 4.5. Effect of pH on the Activity and Stability of MaBGA

The optimum pH for MaBGA was determined by measuring the activity in Britton–Robinson buffer with different pH ranging from 3.0 to 12.0. The pH stability was assayed by measuring the residual activity after incubating MaBGA in different pH buffers at 37 °C for 1 h.

### 4.6. Effect of Temperature on the Activity and Stability of MaBGA

To study the effect of temperature on the activity of MaBGA, the enzyme activity was assayed at different temperatures from 10 to 70 °C with 5 °C intervals at pH 7.0. The thermal stability was determined by assaying the residual activity after incubating the enzyme at 50 °C for 6 h with 1 h intervals.

### 4.7. Effect of NaCl on the Activity and Stability of MaBGA

In order to determine the effects of NaCl on the activity of MaBGA, the enzyme activity was assayed with 0.5, 1, 1.5, 2, 2.5, 3, 3.5, 4, 4.5, 5 M NaCl added individually. The NaCl tolerance of MaBGA was determined by measuring the residual activity after incubating the enzyme in buffers containing diverse concentration of NaCl from 0.5 M to 5M at 37 °C for 1 h.

### 4.8. Effect of Metal Ions and Chemicals on the Activity of MaBGA

To investigate the effects of metal ions and chemicals on the MaBGA activity, 1 mM of KCl, NaCl, FeCl_2_, MnCl_2_, MgCl_2_, CoCl_2_, NiCl_2_, ZnCl_2_, EDTA and 10 mM of l-cysteine, l-glutathion and dithiotreitol were added to the reaction system individually, and the activity of MaBGA was then measured under the standard assay as described above. No chemical was added in the control.

### 4.9. Substrate Specificity

The substrates’ specificity of MaBGA was measured by the standard assay, except that ONPG was replaced by *p*-nitrophenyl-β-d-galactopyranoside, *p*-nitrophenyl-β-d-glucopyranoside, *p*-nitrophenyl-β-d-xylopyranoside, *p*-nitrophenyl-β-d-lactopyranoside, *p*-nitrophenyl-β-d-glucuronide, *p*-nitrophenyl-a-d-galactopyranoside, *p*-nitrophenyl-β-l-arabinopyranoside, *p*-nitrophenyl-β-d-cellobioside, respectively.

### 4.10. Steady-State Kinetic Analysis

For steady-state kinetic analysis, the activity of MaBGA was measured by using various concentrations of ONPG from 0.1 mM to 19 mM. The kinetic constants of the enzyme were determined by using a nonlinear fitting of the Michaelis–Menten equation: *v* = *V*_max_·[*S*]/(*K_m_* + [*S*]), where [*S*] and *K_m_* are the concentration and Michaelis constants of ONPG, respectively.

### 4.11. Linkage Selectivity Analysis

To determine the activity of MaBGA toward different linkage types, Galβ1–3GlcNAc, Galβ1–4GlcNAc and Galβ1–6GlcNAc were used as substrates, respectively. The reaction products were filtered by nitrocellulose membrane with pore size of 0.22 μm, in advance of being subject to detection by HPLC equipped with an Aminex HPX-87P column and differential detector. The column temperature and flow rate were set as 85 °C and 0.5 mL·min^−1^.

### 4.12. Gene Cloning and Sequence Analysis

The partial sequence of gene *mabga* was amplified by using degenerate primer pairs F1/R1, F1/R2, F1/R3, F2/R1, F2/R2, F2/R3, F3/R1, F3/R2, F3/R3, A208/B1, A208/C1, A208/C2, A76/B1, A76/C1, A76/C2, A195/B1, A195/C1 and A195/C2 (Table 1), respectively, which were designed according to the conservative regions of the protein sequence of β-galactosidases. PCR was performed as follows: 95 °C for 4 min; followed by 30 cycles of 95 °C for 1 min, 50 °C for 1 min, and 72 °C for 2 min; with a final extension at 72 °C for 10 min. The amplified fragment was inserted into the pMD18-T vector and then transformed into *E. coli* DH5α for sequencing.

The 5′ and 3′ flanking regions of the known partial sequence were obtained by using DNA Walking *SpeedUp™* Kit of Seegene, which adopted the thermal asymmetric interlaced PCR (TAIL-PCR) strategy [39]. The nested specific primers for upstream and downstream regions were designed based on the obtained partial sequence (Table 5). TAIL-PCR was performed as described by the kit. The amplified fragments were purified and ligated into the pMD18-T vector for sequencing. The upstream, downstream and obtained partial sequences were assembled to obtain a full-length *mabga* gene.

Homologous search in GenBank was performed using the BLAST server (http://www.ncbi.nlm.nih.gov/BLAST). Alignment of multiple protein sequences was conducted using the Clustal X 2.0 program [40] and rendered by ESPript [41]. A phylogenetic tree of multiple β-galactosidase was constructed using the neighbor-joining method [31] in MEGA6 [32], with a bootstrap test of 1000 replicates.

### 4.13. Homology Modelling and Molecular Docking Analysis

The three-dimensional model of MaBGA was constructed by using MODELLER [27], Robetta [26], I-TASSER [28] and SWISS-MODEL [25], respectively. Precise evaluation of the model quality was performed using ProSA-web [29] and PROCHECK [30]. To remove steric clashes, the constructed model was subject to an energy minimization process in vacuum by using the steepest descent method for about 5000 iterations in GROMACS 4.5 [33].

The refined model was used for docking with Galβ1–3GlcNAc, Galβ1–4GlcNAc and Galβ1–6GlcNAc, respectively, using Autodock 4.2 [34] with default parameters. The representation of the protein structure was achieved using the program PyMOL (The PyMOL Molecular Graphics System, Version 1.7 Schrödinger, LLC., New York, NY, USA).

## 5. Conclusions

In this work, a thermostable β-1,3-galactosidase MaBGA derived from *Marinomonas* sp. BSi20414, was first purified to homogeneity and characterized extensively. MaBGA displayed robust stability against heat and strict substrate specificity toward both the glycosyl group and the linkage type. Although further experiments are required to decipher its substrate recognition mechanism, our study provided an attractive alternative for biosynthesis of galacto-oligosaccharide with β1–3 linkage and laid the groundwork for the protein engineering to modify the linkage preference of β-galactosidase.

## Figures and Tables

**Table 1:** Purification of MaBGA.

Purification Steps	Total Protein (mg)	Total Activity (U)	Specific Activity (U/mg)	Recovery (%)	Purification Fold
Cell lysate	162.54	1818.18	11.19	100	1
Ammonium sulfate precipitation	29.9	927.27	31.01	51	2.77
HiTrap DEAE FF	1.01	91.80	90.89	9.9	8.12

**Table 2:** Effects of metal ions and chemicals on the activity of MaBGAL.

Metal Ions	Relative Activity (%)	Chemicals	Relative Activity (%)
K^+^	96	EDTA	98
Na^+^	95	l-Cysteine	110
Fe^2+^	211	l-Glutathion	103
Mn^2+^	98	Dithiotreitol	106
Mg^2+^	89		
Co^2+^	88		
Ni^2+^	76		
Zn^2+^	74		

**Table 3:** Substrate specificity of MaBGA.

Substrate	Relative Activity (%)
*o*-nitrophenyl-β-d-galactopyranoside	100
*p*-nitrophenyl-β-d-galactopyranoside	422
*p*-nitrophenyl-β-d-glucopyranoside	<1
*p*-nitrophenyl-β-d-xylopyranoside	<1
*p*-nitrophenyl-β-d-lactopyranoside	<1
*p*-nitrophenyl-β-d-glucuronide	<1
*p*-nitrophenyl-a-d-galactopyranoside	<1
*p*-nitrophenyl-β-l-arabinopyranoside	<1
*p*-nitrophenyl-β-d-cellobioside	<1

**Table 4:** Evaluation of models generated by different modeling approaches.

Model	*Z*-Score ^1^	Ramachandran Plot ^2^			
		Most Favored (%)	Additional Allowed (%)	Generously Allowed (%)	Disallowed (%)
Template (4oif)	−12.19	88.3	10.7	0.6	0.3
Robetta	−10.19	87.9	10.2	1.6	0.4
Template (1kwk)	−12.24	90.6	8.2	0.7	0.4
SWISS-MODEL	−9.93	89.7	8.1	1.5	0.7
MODELLER	−10.08	91.9	6.8	0.4	0.9
Template (1kwg)	−12.27	91.2	7.9	0.6	0.4
I-TASSER	−10.14	79.1	15.8	3.2	1.9

^1^ Calculated by ProSA-web; ^2^ Calculated by PROCHECK.

**Table 5:** Primers used for gene cloning.

Primers	Sequence (5′ to 3′)	Purpose
F1	GCNTGGGGNAAYGTNTTYT	Degenerated PCR
F2	TNTGGACNTGGGARGCNTT	Degenerated PCR
F3	GGARCARCARCCNGGNCCNGT	Degenerated PCR
R1	CCARCANGCRTCRTARTCRAA	Degenerated PCR
R2	RAANGCYTCCCANGTCCA	Degenerated PCR
R3	GGRTTRTGNGGNGCCARTT	Degenerated PCR
A208	TGGATHATGGAGGAGCCC	Degenerated PCR
A76	CGGGACCTGGTGCAYAAYTAY	Degenerated PCR
A195	CAYAAYTAYATGGGCTTCTTC	Degenerated PCR
B1	CAGACCCAGAACGAGTAYKGN	Degenerated PCR
C1	GCACCACAAGTACCACCARGA	Degenerated PCR
C2	GTYCTRDWNCTGCACCGGCCG	Degenerated PCR
U1	CCGTAAAGAATCCCATGAGT	DNA Walking (1st-round upstream)
D1	GGACATTTTGCGTGCG	DNA Walking (1st-round downstream)
U2	AACGCTGAAAGTCCAACCCGAT	DNA Walking (2nd-round upstream)
D2	GGACACTTATCCGCTGGGTTT	DNA Walking (2nd-round downstream)
U3	GATTGGCTTCGGTCACGGT	DNA Walking (3rd-round upstream)
D3	CCCGATTTTGGTGCTTTTCA	DNA Walking (3rd-round downstream)
DW-ACP 1	ACP-AGGTC	DNA Walking (1st-round)
DW-ACP 2	ACP-TGGTC	DNA Walking (1st-round)
DW-ACP 3	ACP-GGGTC	DNA Walking (1st-round)
DW-ACP 4	ACP-CGGTC	DNA Walking (1st-round)
DW-ACP N	ACPN-GGTC	DNA Walking (2nd-round)
Uni-primer	TCACAGAAGTATGCCAAGCGA	DNA Walking (3rd-round)
MaBGA-F	CGGAATTCAAGTTAGGTGTATGTTACTACCCAG	Full-length amplification
MaBGA-R	GTTCGCGCTCGAGGATTTCTTGCCAAATGGC	Full-length amplification
